# Estrogen Therapy and Ischemic Stroke in Women with Diabetes Aged Over 55 Years: A Nation-Wide Prospective Population-Based Study in Taiwan

**DOI:** 10.1371/journal.pone.0144910

**Published:** 2015-12-14

**Authors:** Yi-Hsin Chen, Teng-Fu Hsieh, Ching-Chih Lee, Ming-Ju Wu, Yun-Ching Fu

**Affiliations:** 1 Institute of Clinical Medicine, National Yang-Ming University School of Medicine, Taipei, Taiwan; 2 Department of Nephrology, Taichung Tzu Chi Hospital, Buddhist Tzu Chi Medical Foundation, Taichung, Taiwan; 3 Department of Urology, Taichung Tzu Chi Hospital, Buddhist Tzu Chi Medical Foundation, Taichung, Taiwan; 4 School of Medicine, Tzu Chi University, Hualian, Taiwan; 5 Community Medicine Research Center and Institute of Public Health, National Yang-Ming University, Taipei, Taiwan; 6 Department of Otolaryngology, Dalin Tzu Chi Hospital, Buddhist Tzu Chi Medical Foundation, Chiayi, Taiwan; 7 Center for Clinical Epidemiology and Biostatistics, Dalin Tzu Chi Hospital, Buddhist Tzu Chi Medical Foundation, Chiayi, Taiwan; 8 Division of Nephrology, Department of Internal Medicine, Taichung Veterans General Hospital, Taichung, Taiwan; 9 School of Medicine, Chung Shan Medical University, Taichung, Taiwan; 10 School of Medicine, College of Medicine, China Medical University, Taichung, Taiwan; 11 Section of Pediatric Cardiology, Department of Pediatrics, Taichung Veterans General Hospital, Taichung, Taiwan, Republic of China; VU University Medical Center, NETHERLANDS

## Abstract

This study explores the possible association between the risk of ischemic stroke and conjugated equine estrogen (CEE) use in women who are over 55 years old and have diabetes. Data from the National Health Insurance system of Taiwan were used to identify 428 women over 55 years old with diabetes who used CEE (0.625 mg daily) from 2003 to 2009. For comparison, 21026 women with diabetes who were from the same cohort and did not use estrogen were used as a control group, excluding patients with previous ischemic stroke at the baseline. The propensity score method was used to identify a 1:3 ratio for the matched cohort (n = 1284). Covariates used for propensity score-matching included age and comorbidities. Cox’s proportional hazard model was applied to estimate the relationship between CEE use and ischemic stroke. The overall incidence of ischemic stroke was significantly lower in patients using CEE than in the control group (0.9% compared with 3.0%, *p* = 0.016). Further analyses using Cox’s proportional hazard model revealed that after adjusting for age, comorbidities, socioeconomic status, urbanization, and other medications associated with ischemic stroke, a lower risk was present in patients with CEE use (hazard ratio: 0.34; 95% confidence interval: 0.12–0.97). Time of menopause could not be identified because of the nature of the database. CEE might decrease the risk of ischemic stroke in women with diabetes aged over 55 years, according to this population-based study.

## Introduction

Prevalence of stroke differs with sex; [1–4] after age 55, 1 in 5 women and 1 in 6 men develop a stroke [5]. An elevated incidence of stroke is observed in women experiencing premature or early menopause [6]; this has resulted in an increase in an interest in the role of estrogen in stroke development.

Numerous epidemiological and observational studies have demonstrated that endogenous estrogen can protect premenopausal women from stroke [7, 8]. However, the effect of estrogen replacement therapy in postmenopausal women has remained controversial, with various studies reporting conflicting results. Among published reports, estrogen replacement therapy was found to decrease [9–12], increase [13], or have no effect [14–22] on the risk of stroke. A study from the Women's Health Initiative (WHI) reported that estrogen plus medroxyprogesterone acetate can increase the risk of cardiovascular disease [23]; however, these effects were limited to older women without a significantly increased risk of total mortality [24]. Because of ethical considerations, clinical trials of estrogen replacement therapy often recruit healthy subjects, while excluding vulnerable patients [25, 26]. Thus, the interpretation of such results cannot be reliably applied to other groups such as those with diabetes. After adjusting for other risk factors, the risk of ischemic stroke among patients with diabetes was found to be double that of individuals without diabetes [27]. Sex and ethnicity also modify the risk of stroke in patients with diabetes. Women with diabetes have a higher risk of stroke than similarly affected men (hazard ratios [HR]s: 2.8 and 2.2, respectively) [27]. Estrogen has been shown to play a physiological role in protection against cardiovascular disease [28, 29]. Ferrara et al. found that women who had diabetes and had not recently developed a myocardial infarction continued to have a lower risk of a future infarction following estrogen replacement therapy (HR: 0.81; 95% confidence interval [CI]: 0.66–1.00) [30]. These findings suggest that patients with diabetes may experience stroke-preventing benefits from estrogen replacement therapy.

Taiwan’s National Health Insurance Research Database (NHIRD), maintained by the National Health Research Institutes, is a national database covering 26 million administered insured patients. This system covers 99% of the population and provides health care for the entire population of Taiwan. The use of this database therefore provides an advantage in investigating the incidence of diseases and medical intervention outcomes. In this study, we used the NHIRD to investigate incidence of ischemic stroke following estrogen use in women with diabetes aged over 55 years.

## Materials and Methods

### Ethics

This study was conducted after being approved by the Institutional Review Board of the Buddhist Taichung Tzu Chi General Hospital, Taiwan (REC103-43). Because the identification numbers and personal information of individuals included in the study were not used in the secondary files, the Review Board stated that written consent from patients was not required.

### NHIRD database

In 1995, Taiwan initiated a National Health Insurance (NHI) program that requires mandatory enrollment in a government-run, universal, single-payer health insurance system. Currently, nearly 99% of the 23 million residents of Taiwan receive medical care through the NHI program. NHI contracts with over 97% of the hospitals and clinics in Taiwan to provide health care services [31]. All data related to these services are collected and input into the NHIRD by the National Health Research Institutes to provide a comprehensive record of medical care. The database includes ambulatory care records, inpatient care records, and the registration files of insured patients. The National Health Insurance Bureau of Taiwan randomly reviews the charts of 1 out of every 100 ambulatory cases and 1 out of every 20 inpatient cases, additionally performing patient interviews to verify the accuracy of diagnoses [32]. This study used a subset of the NHIRD containing comprehensive healthcare data regarding the ambulatory care claims, inpatient claims, and prescriptions of 1,000,000 people randomly selected among all insured beneficiaries.

This study used NHIRD data obtained from January 1, 2003 to December 31, 2009, as published by Taiwan’s National Health Research Institutes. Patients with diabetes (n = 109542) were identified from the dataset.

### Estrogen and control group selection

**Fig 1:**
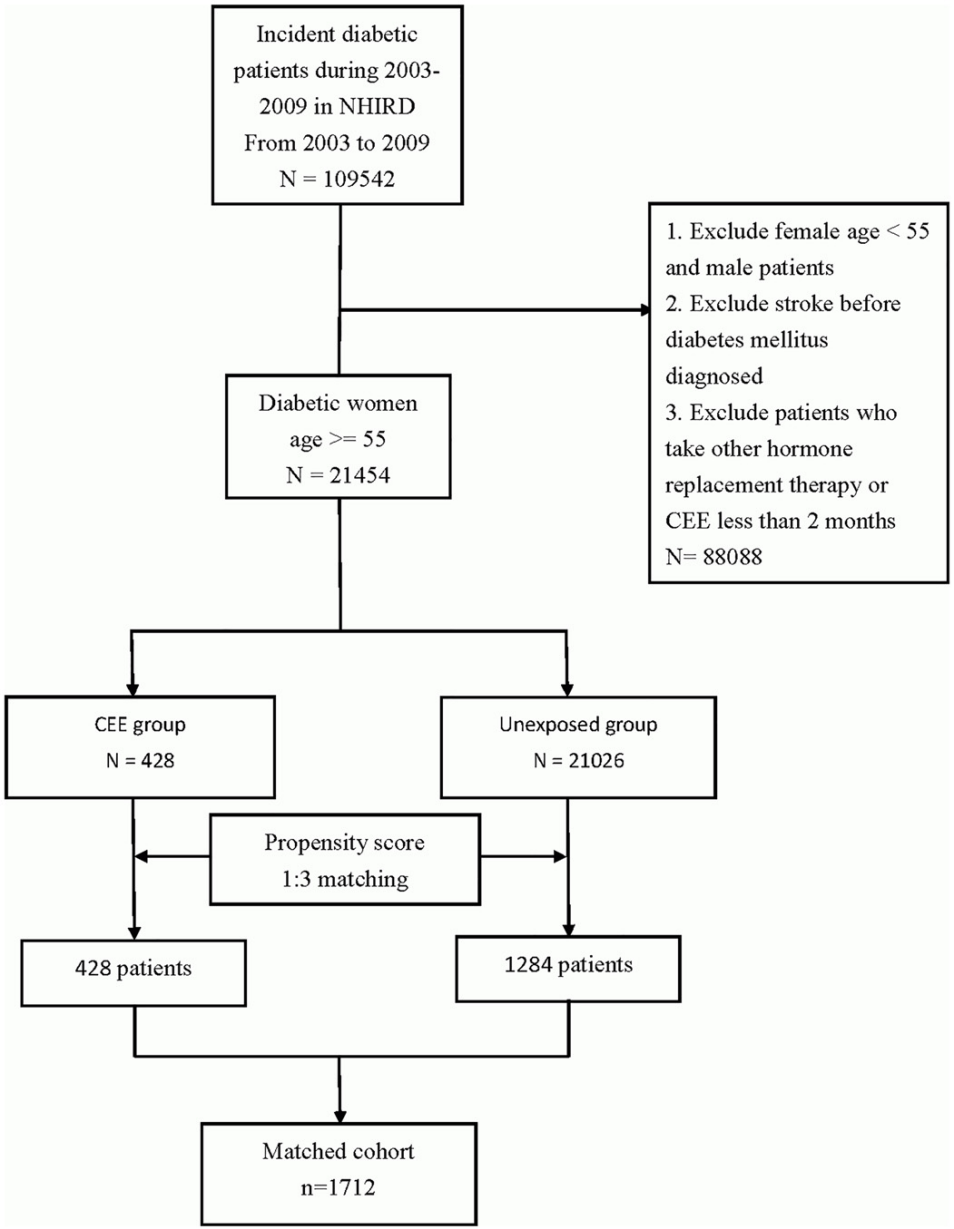
Flow chart depicting the inclusion criteria for each group of patients. CEE, conjugated equine estrogen; NHIRD, National Health Insurance Research Database.

Patients were selected for inclusion in the study group (n = 428) if they were prescribed 0.625 mg daily conjugated equine estrogen (CEE) and met the following criteria: (1) were women at least 55 years old during the enrollment interval (between January 1, 2003 and December 31, 2009) [33–36], (2) had a diagnosis of diabetes (according to the International Classification of Disease, Ninth Revision, Clinical Modification [ICD-9-CM] codes 250.X), (3) orally used CEE for at least 60 days within 3 continuous months during the enrollment interval; and (4) had no exposure to any other oral estrogen prior to enrollment. Patients who experienced previous strokes (ICD-9-CM codes 430–438) and atrial fibrillation (ICD-9-CM codes 427.31) before diabetes mellitus was diagnosed were excluded. Validation of these diagnoses was ensured by verification of at least 3 separate outpatient visits by the same individual. A control group (n = 21026) was selected from diabetic women over 55 years old who did not use any estrogen therapy during the study period in the database. A propensity score-matching approach was used to create a subgroup for further adjustment of potential selection bias between the estrogen-using and control groups [37]. Propensity score matching is a method used in this case to control for potential confounding variables by balancing covariates between groups of patients who did or did not receive exposure to CEE. A propensity score regarding the probability of receiving CEE was calculated for each patient using a logistic regression model including the covariates of age and comorbidities (including hypertension, hyperlipidemia, chronic kidney disease, coronary artery disease, and heart failure). The propensity score was then used to match patients in the estrogen-using and control groups at a ratio of 1:3 via the propensity score nearest-neighbor matching method. The randomly selected patients in the CEE group were matched to the patients in the control group who had the closest propensity score within a score width of 0.01. This process was repeated until 1284 patients matched by propensity score from the control group (n = 21026) were found. Patients were excluded from the cohort analysis if no match was found. Based on these criteria, a matched cohort with 1712 persons was included, as shown in Fig 1. The endpoint of the study was defined as the first recorded inpatient claim of ischemic stroke (ICD-9-CM Codes 433–438).

### Variables

The independent variables examined in the study included age; comorbidities of hypertension (ICD-9-CM Codes 401–405), hyperlipidemia (ICD-9-CM Code 272), coronary artery disease (CAD; ICD-9-Codes 410–414), heart failure (ICD-9-CM Code 428), and chronic kidney disease (ICD-9-CM Code 585); urbanization; and socioeconomic status (SES). SES was classified into two categories: low SES (less than $20000 NT, equivalent to 625 United States dollars [USD] per month), and high SES (at least $20001 NT, equivalent to 626 USD per month).

### Statistical analysis

SPSS 15 software was used for data analysis. Pearson’s chi-squared test was applied to categorical variables such as sex, SES, area of residence, and comorbidities. Continuous variables were analyzed using a one-way ANOVA test. The cumulative risk of ischemic stroke for patients with and without estrogen use was estimated using Kaplan-Meier survival curves. Cox’s proportional hazard model, adjusted for patient characteristics (including age, co-morbidities, SES, and geographic regions), was used to analyze the association of CEE use with the subsequent risk of ischemic stroke during the 5-year follow-up period. We calculated HR and 95% CI values using a significance level of 0.05. A two-sided *p*-value (*p* < 0.05) was used to determine statistical significance.

## Results

### Patient characteristics

In total, 21,454 patients were included in our study cohort. Of these individuals, we identified 428 patients who used CEE and 21,026 patients who had no history of such use. A matched cohort (based on the propensity score) including 428 and 1284 patients in the estrogen-using and control groups (at a ratio of 1:3), respectively, was identified. All characteristics accounted for in both groups were comparable following application of the matching criteria. The demographic characteristics and selected comorbidities for the two groups are shown in Table 1. Both groups had similar age distributions (with a median age of 59 years for both groups; Table 1).

**Table 1:** Baseline characteristics (n = 1712).

Characteristics	With CEE	Control	*p*-value
Total number of patients	428	1284	
Median age in years (range)	59 (55–90)	59 (55–91)	0.715
Age group			0.932
55–64	332(77.6)	988(76.9)	
65–75	83(19.4)	259(20.2)	
Over 75	13(3.0)	37(2.9)	
Coronary artery disease	30 (7.0)	94 (7.3)	0.830
Heart failure	2 (0.5)	13 (1.0)	0.295
Patients using other drugs:			
Aspirin	125 (29.2)	393 (30.6)	0.585
Clopidogrel	26 (6.1)	75 (5.8)	0.859
SES:			0.911
Low SES	205 (47.9)	611 (47.6)	
High SES	223 (52.1)	673 (52.4)	
Urbanization:			0.111
Urban	105 (24.5)	366 (28.5)	
Rural	323 (75.5)	918 (71.5)	
Geographic region:			0.645
Northern	270 (63.1)	794 (61.8)	
Southern	158 (36.9)	490 (38.2)	

Age is given as the median (range). Other values are the number of patients (percentage), as indicated. *t*-test was used. Abbreviations: CEE, conjugated equine estrogen; SES, socioeconomic status.

### Relation of estrogen use and stroke incidence

**Fig 2:**
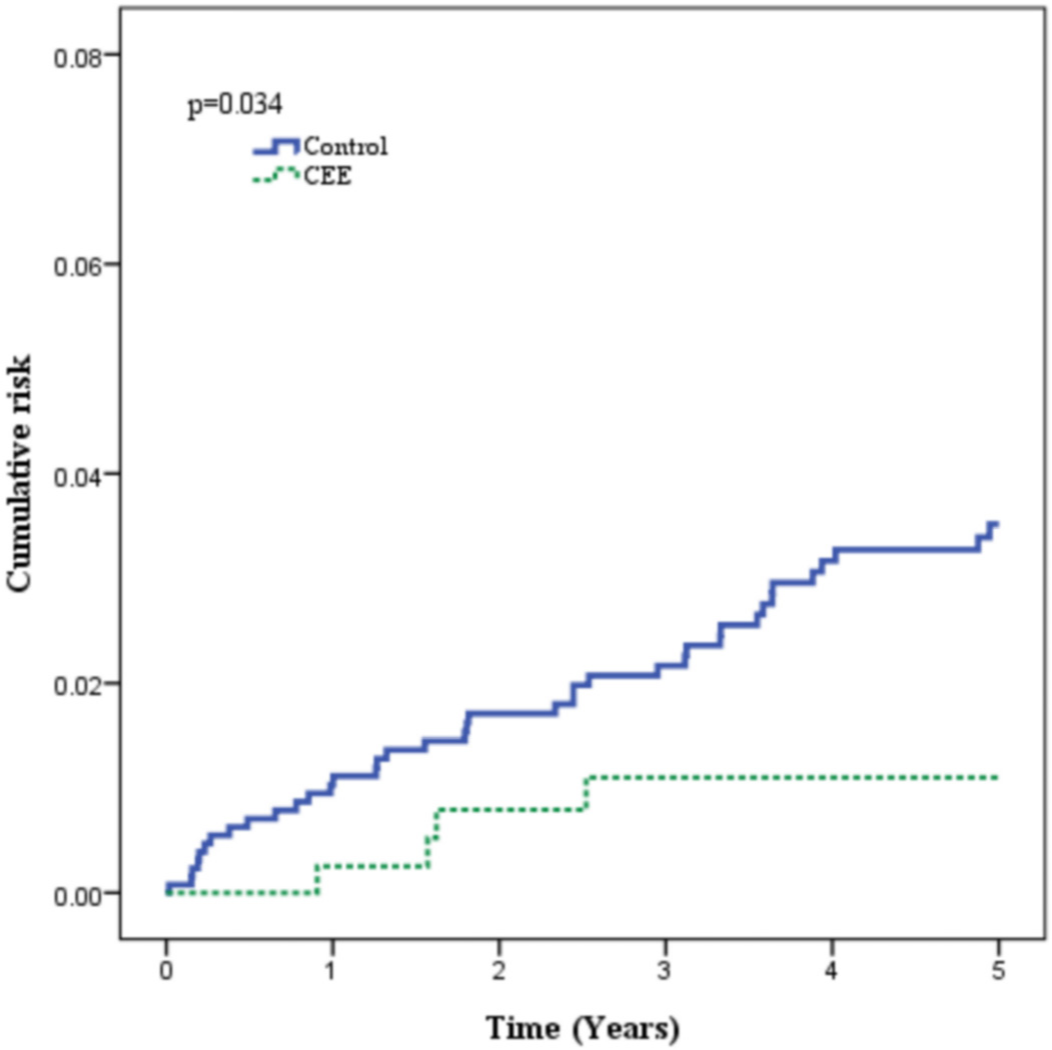
Kaplan-Meier analysis for comparison of cumulative risk for ischemic stroke between conjugated equine estrogen (CEE) and control groups. Data indicate percentage values among each group for which the incidence of ischemic stroke differed significantly (log-rank *p* = 0.034).

Fig 2 shows the Kaplan-Meier failure curve depicting ischemic stroke incidence in patients with and without CCE use. The patients who used estrogen had significantly fewer events defined as ischemic stroke than those in the control group during the 5-year follow-up period (*p* = 0.034). At the end of the follow-up period, 42 patients had developed ischemic stroke, including 4 individuals (0.9%) in the estrogen group and 39 (3.0%) in the control group (Table 2; *p* = 0.016). For the age subgroups, the frequency of stroke in patients aged less than 65 years in the estrogen group was 0.9% (three stroke cases were found; the ages of the patients were 55, 56, and 64 years), whereas it was 2.4% in the control subgroup of the same age. In the subgroup with patients aged between 65 and 75 years, the stroke incidence in the estrogen group was 1.2%(one stroke case was found; the age of the patient was 66 years), whereas it was 4.7% in the control subgroup of the same age. There were no stroke cases in the estrogen group in the subgroup aged greater than 75 years.

**Table 2:** Cumulative rate of stroke over 5 years in diabetes patients with and without estrogen.

Characteristics	Number of patients	Events (%)	*p*-value
Estrogen status:			*0*.*016*
CEE	428	4 (0.9)	
Control	1284	39 (3.0)	

Abbreviations: CEE, conjugated equine estrogen.

After correcting the model for the effects of age, comorbidities (hypertension, hyperlipidemia, chronic kidney disease, coronary artery disease, and heart failure), and medications (aspirin or clopidogrel), the CEE group exhibited a significantly lower risk of ischemic stroke (Table 3; HR: 0.34, 95% CI: 0.12–0.97) than the control group. The median duration of exposure in the CCE group was 6.5 months (with a range of 2–60 months). The hysterectomy status of patients could not be reliably ascertained in this data set, as surgical procedures prior to 2003 were not included.

**Table 3:** Multivariate-adjusted stroke hazard ratios among patients with diabetes with or without CCE use over a 5-year period.

Variable	5-year stroke risk		
	Adjusted HR*	95% CI**	*p*-value
Group:			
Control	1		
CEE	0.34	0.12–0.97	0.045
Drug:			
Aspirin	1.02	0.50–2.05	0.956
Clopidogrel	2.50	0.75–6.22	0.538
Comorbidities			
Hypertension	0.84	0.42–1.68	0.624
Hyperlipidemia	0.85	0.33–2.18	0.737
Chronic kidney disease	1.64	0.21–12.51	0.634
Coronary artery disease	0.87	0.29–2.56	0.801
Heart failure	1.89	0.24–14.55	0.539
Demographics:			
Low SES	1		
High SES	0.95	0.51–1.77	0.889
Urbanization:			
Urban	1		
Non-urban	1.71	0.73–3.96	0.209
Geographic Region:			
Northern	1		
Southern	1.75	0.94–3.27	0.075

Abbreviations: CEE, conjugated equine estrogen; SES, socioeconomic status.

*Adjusted HR, adjusted hazard ratio.

**95% CI, 95% confidence interval.

## Discussion

This population-based study revealed a significantly lower incidence of ischemic stroke in women with diabetes aged over 55 years using CEE when compared with women in the control group, who had no exposure to exogenous estrogen.

After adjustment for potential confounding factors, CEE use was associated with a 66% reduction in risk of ischemic stroke compared with the control group. Few studies have specifically demonstrated the effect of estrogen on cardiovascular events in postmenopausal women with diabetes [30]. The results of the present study suggest that estrogen treatment might decrease the incidence of ischemic stroke in diabetic women aged over 55 years.

The present study presents a nation-wide, population-based investigation using a database that is routinely validated for diagnostic accuracy by the National Health Insurance Bureau of Taiwan. The database includes all ambulatory- and inpatient-care records and covers approximately 97% of the total population and hospitals in Taiwan. All residents in Taiwan have equal access to health care, thus providing favorable conditions for epidemiological investigations.

Epidemiological and clinical studies have previously indicated that atrial fibrillation is a major independent risk factor for stroke [38–42]. The Framingham study of atrial fibrillation found that women with overt coronary heart disease were subject to a nearly 5-fold increase in the risk of stroke [42]. Thus, patients with a history of atrial fibrillation were excluded from the present study to eliminate any potential bias in this regard. After adjustment for other covariates, women with diabetes were found to have a higher risk of ischemic stroke (HR: 2.83, CI: 2.35–3.40) [27]. Furthermore, diabetes was previously found to increase the risk of stroke by an extra 40% within the population of patients with atrial fibrillation [43]. Sanne et al. conducted a meta-analysis and found that women with diabetes had a 27% greater risk of stroke compared with the respective population of men [44]. Numerous studies have found that diabetes in women is more likely to be associated with an increase in cardiovascular risk than diabetes in men [45–50]. Women with diabetes are also less likely to receive proper treatment for abnormalities in blood pressure, low-density lipoprotein, fasting glucose, and glycated hemoglobin [51, 52]. Premenopausal women have a lower incidence of cardiovascular disease than postmenopausal women [53, 54]. These findings suggest that estrogen could potentially contribute to stroke prevention, especially within the subpopulation of women with diabetes.

Several experiments have suggested that the mechanism by which estrogen treatment results in vascular protection could be through the activation of eNOS in endothelial cells [55, 56]. Hayashi et al. demonstrated that estrogen treatment can ameliorate endothelial dysfunction caused by hyperglycemia [57], and concluded that estrogen confers protection in patients prone to high blood sugar levels, such as women with diabetes. However, a combination of estrogen and medroxyprogesterone acetate was found to increase the risk of coronary heart disease and stroke in a WHI study [23]. Interestingly, other studies have found that medroxyprogesterone acetate counteracts the atheroprotective effects of estrogen [58–60]. Thus, previous combination therapy studies have suggested a potentially detrimental effect of hormone replacement on cardiovascular protection. Postmenopausal women with diabetes present worse cardiometabolic profiles than women without diabetes and aged-matched men [61]. The causes of stroke in this particularly high-risk group therefore require further investigation. An estrogen-only preparation was specifically chosen in our study to survey the effects on ischemic stroke.

Estrogen has been shown to reduce complications in patients with diabetes in previous studies [62, 63]. Estrogen was also found to have a modulating effect on glucose homeostasis [64, 65]. Based on the results of the aforementioned studies, estrogen may play a role in reducing cardiovascular complications in women with diabetes over 55 years old, which is when the majority of women enter into menopause.

Previous studies have shown that the rare factor V Leiden mutation results in a lower number of cardiovascular events in different ethnic groups [66, 67]. Genetic background should thus be specifically considered when performing studies on different ethnic groups. Because genetic polymorphisms could not be analyzed in our study, we assumed that the genetic backgrounds were equal between the two groups to eliminate bias.

The utilization rates of hormone replacement therapy ranged between 7.00% and 13.10% of women over 40 years old in Taiwan from 2000 to 2004 [68]. However, previous studies have not indicated a detailed distinction between the uses of single or combination hormone therapy within the cohorts investigated. We chose to investigate the use of single hormone (estrogen) therapy in our cohort, and to match patients in the control group to reduce the confounding effects of different doses.

Previous studies have demonstrated that SES, which is correlated positively with the use of estrogen, modifies the risk of cardiovascular disease in postmenopausal women [69]. However, an impact of SES on the incidence of stroke was not found using our application of the multivariate model (Table 3; *p* = 0.889).

Stroke is often related to dementia and coronary artery disease. Further analysis in our dataset revealed that the CEE group had a lower risk of dementia (crude HR: 0.57, CI: 0.40–0.81), but not significantly after adjustment for other covariates (adjusted HR: 1.03, CI: 0.72–1.48). For coronary artery disease, no significant difference was found between the CEE and control groups (crude HR: 0.8, CI: 0.70–1.1; adjusted HR: 0.92, CI: 0.73–1.16).

### Limitations

This study has several limitations. First, the diagnoses of ischemic stroke and any other comorbid conditions were completely based on ICD codes, rather than more stringent criteria. However, validation of these diagnoses was confirmed if more than 3 different outpatient visits were recorded. Furthermore, the NHIB of Taiwan randomly reviews patient data and conducts patient interviews to verify diagnosis accuracy. Hospitals are regularly audited, and fines are levied for malpractice or significant discrepancies. Second, the severity of ischemic stroke cannot be precisely ascertained via ICD codes, which prevents further subgroup analysis. Third, the database does not contain information regarding tobacco use, dietary habits, and body mass index, which may also be risk factors for stroke. Additionally, the precise time of menopause could not be identified because the nature of the database. Time since menopause was an important factor in the secondary analysis of the Women’s Health Initiative study [24], which found that hormone therapy closer to menopause was associated with reduced coronary heart disease risk. However, the risk of stroke since menopause did not change. Our results should be carefully interpreted when considering the time of menopause. Fourth, the incidence of ischemic stroke in the estrogen-using group was low (0.9%). However, the data were drawn from a sample of 1,000,000 NHIRD patients. Previous studies have also reported low stroke incidence rates. For instance, a rate of 0.29% was reported in a group using a combination of estrogen and progestin [23]. Other indications of estrogen use among these patients could not be ascertained by our study; however, we have considered confounding factors to the greatest extent possible, given the available data. Additionally, this study was conducted on women with diabetes aged over 55 years; thus, our findings may not be generalized to other populations. The identification of links between administrative data and primary hospitalization information, including the severity of stroke and other detailed risk factors, merits investigation in future studies.

## Conclusion

Our nation-wide retrospective population-based analysis of estrogen use reveals a lower incidence of ischemic stroke in women with diabetes aged over 55 years. Future randomized clinical trials are merited to confirm these results.
